# Phylogenetic Analysis of a Spontaneous Cocoa Bean Fermentation Metagenome Reveals New Insights into Its Bacterial and Fungal Community Diversity

**DOI:** 10.1371/journal.pone.0038040

**Published:** 2012-05-29

**Authors:** Koen Illeghems, Luc De Vuyst, Zoi Papalexandratou, Stefan Weckx

**Affiliations:** Research Group of Industrial Microbiology and Food Biotechnology (IMDO), Faculty of Sciences and Bio-engineering Sciences, Vrije Universiteit Brussel, Brussels, Belgium; Auburn University, United States of America

## Abstract

This is the first report on the phylogenetic analysis of the community diversity of a single spontaneous cocoa bean box fermentation sample through a metagenomic approach involving 454 pyrosequencing. Several sequence-based and composition-based taxonomic profiling tools were used and evaluated to avoid software-dependent results and their outcome was validated by comparison with previously obtained culture-dependent and culture-independent data. Overall, this approach revealed a wider bacterial (mainly *γ-Proteobacteria*) and fungal diversity than previously found. Further, the use of a combination of different classification methods, in a software-independent way, helped to understand the actual composition of the microbial ecosystem under study. In addition, bacteriophage-related sequences were found. The bacterial diversity depended partially on the methods used, as composition-based methods predicted a wider diversity than sequence-based methods, and as classification methods based solely on phylogenetic marker genes predicted a more restricted diversity compared with methods that took all reads into account. The metagenomic sequencing analysis identified *Hanseniaspora uvarum, Hanseniaspora opuntiae, Saccharomyces cerevisiae, Lactobacillus fermentum*, and *Acetobacter pasteurianus* as the prevailing species. Also, the presence of occasional members of the cocoa bean fermentation process was revealed (such as *Erwinia tasmaniensis*, *Lactobacillus brevis*, *Lactobacillus casei*, *Lactobacillus rhamnosus*, *Lactococcus lactis*, *Leuconostoc mesenteroides*, and *Oenococcus oeni*). Furthermore, the sequence reads associated with viral communities were of a restricted diversity, dominated by *Myoviridae* and *Siphoviridae*, and reflecting *Lactobacillus* as the dominant host. To conclude, an accurate overview of all members of a cocoa bean fermentation process sample was revealed, indicating the superiority of metagenomic sequencing over previously used techniques.

## Introduction

Cocoa beans are seeds embedded in a mucilaginous pulp in fruit pods of the cocoa tree, *Theobroma cacao* L., and are used as the basic raw material for chocolate production [1], [2]. The desired characteristic cocoa flavor and taste is obtained by fermenting, drying, and roasting of the raw cocoa beans [3], [4]. The first step in cocoa processing is a spontaneous three- to six-day fermentation of the cocoa pulp-bean mass, in most cases carried out in heaps or boxes, wherein a succession of microbial activities of yeasts, involved in depectinization and ethanol formation, lactic acid bacteria (LAB), involved in citric acid fermentation and lactic acid production, and acetic acid bacteria (AAB), involved in the oxidation of ethanol produced by the yeasts into acetic acid and overoxidation of acetic acid and of lactic acid produced by LAB into carbon dioxide and water, takes place [5]–[7]. During fermentation, ethanol and acetic acid diffuse into the beans, and this, in combination with the heat produced during fermentation in general and during ethanol oxidation in particular, causes the death of the seed embryo. This step in turn initiates physical and biochemical changes in the beans, leading to the formation of precursor molecules for the development of a characteristic flavor and color of well-fermented cocoa beans [8]–[10].

During the last decade, the microbial diversity of spontaneous cocoa bean fermentation processes has been investigated through the application of culture-dependent and culture-independent techniques [11]–[22]. This has resulted in a better knowledge of this peculiar microbial ecosystem, which is dominated by species such as *Hanseniaspora* sp., *Saccharomyces cerevisiae*, *Lactobacillus fermentum*, *Lactobacillus plantarum*, and *Acetobacter pasteurianus*. However, it is known that both approaches have some drawbacks, undermining an accurate view on the microbial composition of this ecosystem, and implying that more, yet unidentified species, might play a role in the fermentation process. For instance, it has been shown that culture-dependent techniques can enhance the recovery of certain species that are not necessarily the most abundant or important ones in an ecosystem, thereby giving a non-accurate quantitative view [23]. To circumvent this drawback, culture-independent techniques such as denaturing gradient gel electrophoresis of small PCR amplicons of the targeted gene fragments (PCR-DGGE) or rRNA gene clone library sequencing have been used, also in the case of cocoa bean fermentation processes [7], [14]. These techniques aim at the identification of both cultivable and yet uncultivable but potentially important players in a microbial ecosystem in a semi-quantitative way, thereby using whole microbial community (metagenomic) DNA. However, these methods might give a biased outcome for several reasons too, as they rely on PCR, thereby suffering from typical artifacts such as preferential DNA amplification. [24]. Moreover, PCR-DGGE is based on the amplification of several, rather small, variable regions of mostly the 16S (bacteria) or 26S rRNA genes (yeasts), of which the resolution within some genera is limited [25]–[27]. Recently, 454 pyrosequencing has been used to investigate the bacterial communities by sequencing of 16S rRNA gene amplicons solely [28], [29]. This has also been done for fermented foods, such as nukadoko and kefir [30], [31]. However, as the same short variable regions of the 16S rRNA genes as for PCR-DGGE are targeted, these gene fragments limit this approach.

**Table 1:** Statistics on the environmental reads of two GS FLX Titanium pyrosequencing runs of the metagenomic DNA of a Brazilian spontaneous cocoa bean box fermentation sample.

Statistical parameter	Data set A	Data set B
Number of reads	456,225	1,248,151
Total number of bases	200,550,104	551,635,450
Mean read length	439.08	441.96
Median read length	495	492
% G+C	49.74	49.54

Whole-community sequence data, obtained by high-throughput parallel sequencing of metagenomic DNA, overcome the limitations of the aforementioned culture-dependent and culture-independent techniques [32]. Concerning industrial fermentations involving bacteria, 454 pyrosequencing has recently been applied for assessing the prokaryotic community composition and functionality of, among others, a biogas fermentation process [33] and a kimchi fermentation process [34]. In the area of eukaryotic metagenomics, only a few studies involving whole-community pyrosequencing have been performed, with a focus on fungal diversity associated with soil and plants [35], [36]. To our knowledge, the metagenomic approach has never been used to identify the members of a microbial ecosystem consisting of both prokaryotic and eukaryotic microorganisms, such as in the case of cocoa bean fermentation processes. Yet, to perform such taxonomic profiling, several computational methods are available, tackling either a composition-based [37]–[39] or a similarity-based [40]–[42] approach. It is unclear which of these methods result in the best estimate of microbial diversity. Indeed, similarity-based methods will only be accurate if a close evolutionary relative of a generated sequence (read) is present in the database [43] and these methods are known to be computationally expensive [40]. In the case of (supervised) composition-based methods, it is (often incorrectly) assumed that the genomes available in public databases are representative for the microorganisms present in the ecosystem [44]. Also, these methods can suffer from robustness when short sequences (<1 kb) are used [40].

**Figure 1:**
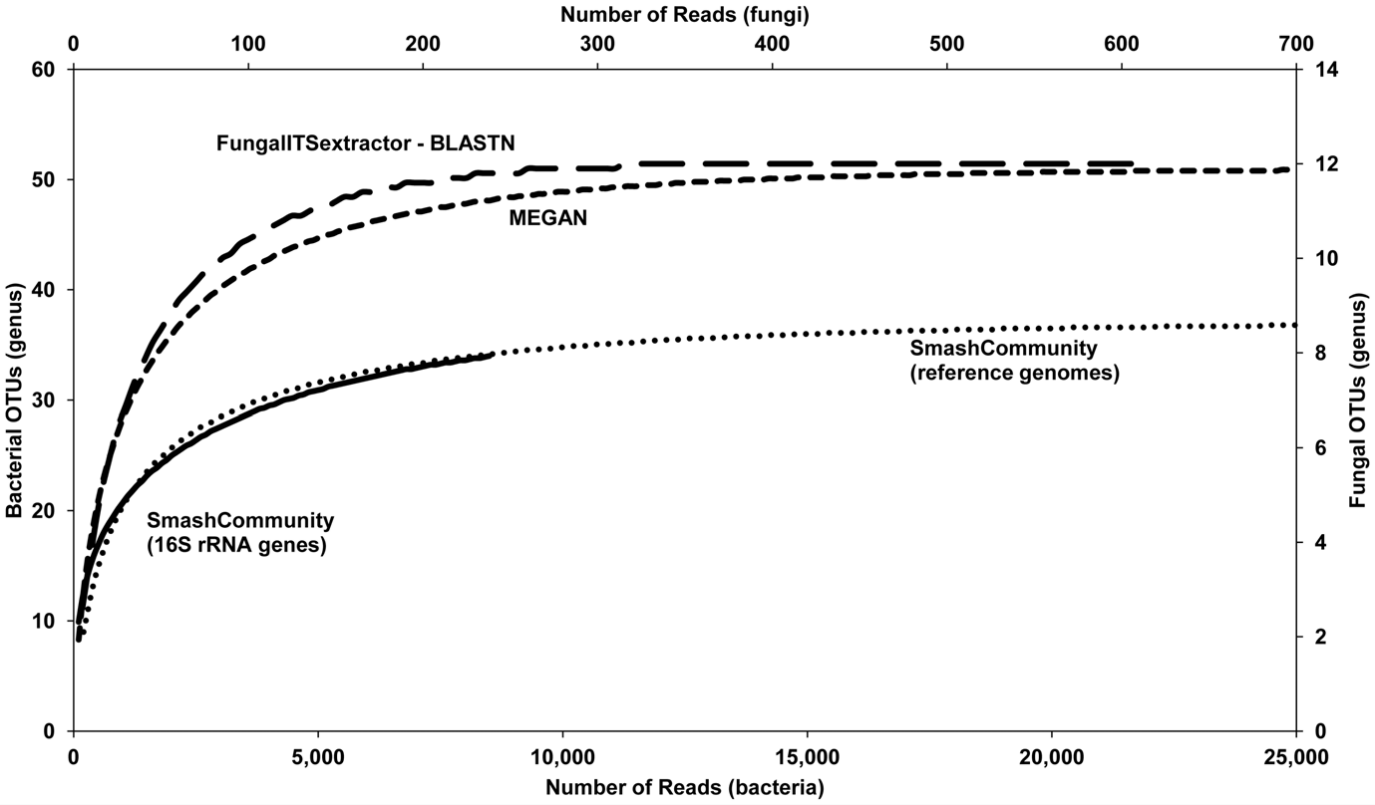
Rarefaction analysis of the genera found with data sets A and B. The rarefaction curves represent an estimation of the number of genera associated with different sampling sizes. As the results of the two 16S rRNA gene-based methods of the SmashCommunity platform were similar, only one method (based on similarity with the 16S rRNA gene sequence database of the SmashCommunity platform) is shown. As the plateau phase of the SmashCommunity reference genomes platform and MEGAN was reached at 25,000 reads, the X-axis is limited to this number of reads.

The aim of the present study was to investigate the microbial communities of a single sample of a spontaneous cocoa bean box fermentation process by performing 454 pyrosequencing on metagenomic DNA, and to compare the outcome with previous data of this sample to validate this metagenomic approach. [7], [14]. Further, using these data, both similarity-based and composition-based computational methods for taxonomic profiling were evaluated and only operational taxonomic units (OTUs) that were consistently predicted were taken into account to avoid a software-dependent outcome. Hence, a complete and more reliable insight into the microbial diversity of the sample studied could be obtained. The results showed that 454 pyrosequencing can be used to identify the bacterial and fungal community members and to provide an insight into the viral communities of a cocoa bean fermentation sample. Analysis of bacterial diversity with multiple taxonomic profiling tools revealed differences in diversity estimates and abundance, which were consistent on different taxonomic ranks. Overall, a wider community diversity was retrieved compared with previous methods, indicating the superiority of metagenomic sequencing.

## Materials and Methods

### Total community DNA preparation, pyrosequencing, and sequence data quality control

A spontaneous cocoa bean box fermentation was performed at the ‘Leão De Ouro’ plantation in Ilhéus (Bahia, Brazil), as described previously [14]. A sample of 500 g was taken 30 h after the start of the fermentation, as at this time point, LAB and AAB species start to control the fermentation, while yeast species, involved during the first hours of the fermentation, are still present [14]. Whole-community metagenomic DNA was isolated in triplicate, each time from 20 g of the sample, as described previously, with minor modifications [12]. Briefly, a NucleoSpin column (Macherey Nagel GmbH, Düren, Germany) was used to remove cocoa pulp compounds, such as polysaccharides, proteins, enzymes, and polyphenols [45]. Furthermore, a second isopropanol precipitation step was applied after RNase treatment, to obtain pure high-quality DNA. The three DNA extracts were pooled and used as template for shotgun pyrosequencing on a Genome Sequencer (GS) FLX system (Roche Applied Science, Mannheim, Germany) using Titanium chemistry, which was performed by the VIB Nucleomics Core Facility (Leuven, Belgium). A DNA library was constructed according to the GS FLX Rapid Library Preparation Kit (Roche Applied Science). The optimal DNA copy per bead ratio was determined by an emulsion PCR titration using a GS FLX Titanium SV emPCR kit (Lib-L; Roche Applied Science). Final emulsion PCR for sequencing production runs was performed using the GS FLX Titanium LV emPCR kit (Lib-L; Roche Applied Science). Two independent pyrosequencing runs were carried out with this DNA library, the first one using two regions of a four-region gasket (half a PicoTiterPlate, the reads were represented by data set A) and the second one using a complete PicoTiterPlate (data set B). To assess the overall comparability of the sequence data sets, average G+C contents of all reads were determined for each data set. Therefore, various Perl scripts were developed to determine the overall and individual G+C contents of the reads. Artificially created duplicate reads were assessed using the Bioconductor software package ShortRead 1.6.2 [46] and cd-hit-454 [47].

**Figure 2:**
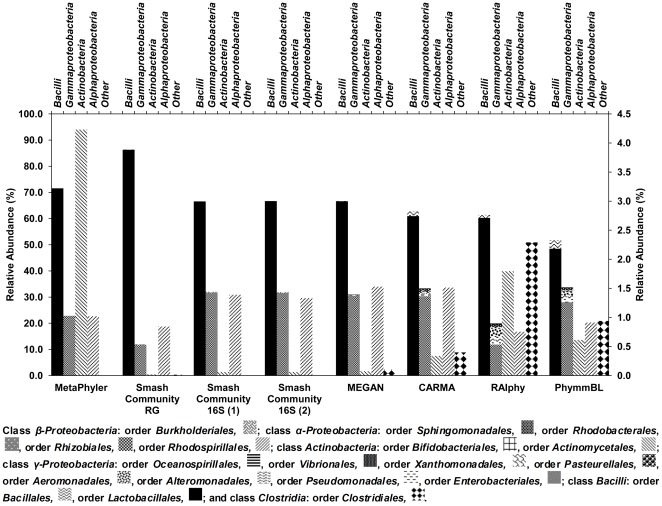
Bacterial composition analysis on ranks class and order by using different taxonomic profiling tools. Classes within the orders *Bacilli* and *γ-Proteobacteria* are shown on the left y-axis; classes within the orders *Actinobacteria*, *α-Proteobacteria*, and others are shown on the right y-axis. ‘SmashCommunity RG’ depicts SmashCommunity reference genomes, ‘SmashCommunity 16S (1)’ depicts the SmashCommunity 16S rRNA gene-based method using the meta_rrna approach, ‘SmashCommunity 16S (2)’ depicts the SmashCommunity 16S rRNA gene-based method using the 16S rRNA gene sequence database approach.

**Figure 3:**
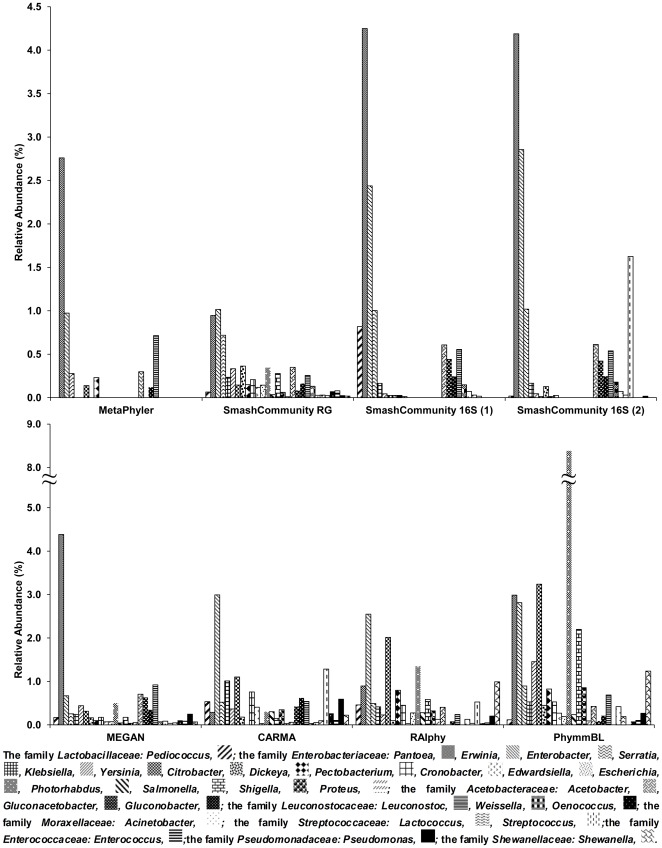
Bacterial composition analysis on rank genus of the low-abundance members by using different taxonomic profiling tools. ‘SmashCommunity RG’ depicts SmashCommunity reference genomes, ‘SmashCommunity 16S (1)’ depicts the SmashCommunity 16S rRNA gene-based method using the meta_rrna approach, ‘SmashCommunity 16S (2)’ depicts the SmashCommunity 16S rRNA gene-based method using the 16S rRNA gene sequence database approach.

### Bacterial and fungal community richness estimation through rarefaction analysis

Binning of the reads for bacterial and fungal community richness estimation through rarefaction analysis was performed on rank ‘genus’ for data set A as well as for the combined data sets A and B. For bacterial rarefaction analysis, several similarity-based classification tools were used, including tools based on phylogenetic marker genes solely as well as tools that took all environmental reads into account. For the phylogenetic marker gene-based binning approach, SmashCommunity (version 1.5) [48] was used to extract 16S rRNA gene sequence fragments from the data sets. Therefore, two binning approaches (based on either a 16S rRNA gene database or on recognition by the meta_rrna tool) supported by this platform were used for detection of the 16S rRNA gene sequences, both using default parameters. This was followed by their classification with the Ribosomal Database Project (RDP) classifier using default parameters. The binning approach based on all environmental reads was performed with SmashCommunity and MEGAN (version 4.40.5) [42]. SmashCommunity was used to align all metagenomic reads to reference genomes through a BLASTN-based sequence similarity search of a SmashCommunity-compatible reference genome database (microbial reference genomes version 2.0; http://www.bork.embl.de/software/smash/). MEGAN was used with the min support set to 100, the min score set to 100, and the top percent set to 7. Hereto, all reads were aligned to the NCBI-nr database (National Center for Biotechnology Information, Bethesda, Maryland, USA) using the BLASTX algorithm.

**Figure 4:**
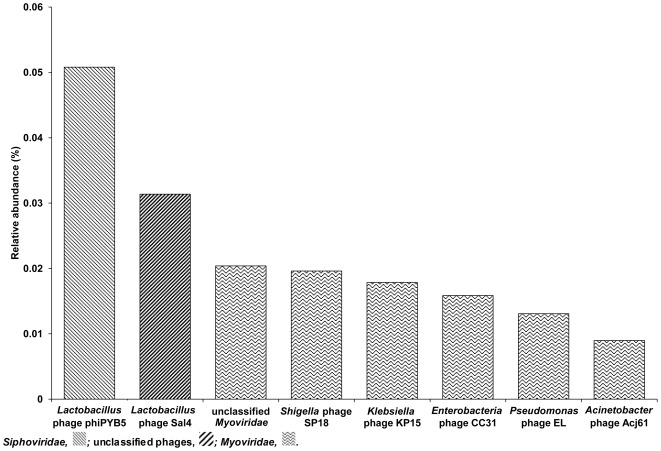
Reads classified as bacteriophages by MEGAN analysis on rank ‘species’. The y-axis depicts the relative abundance compared to the total reads assigned by MEGAN.

**Figure 5:**
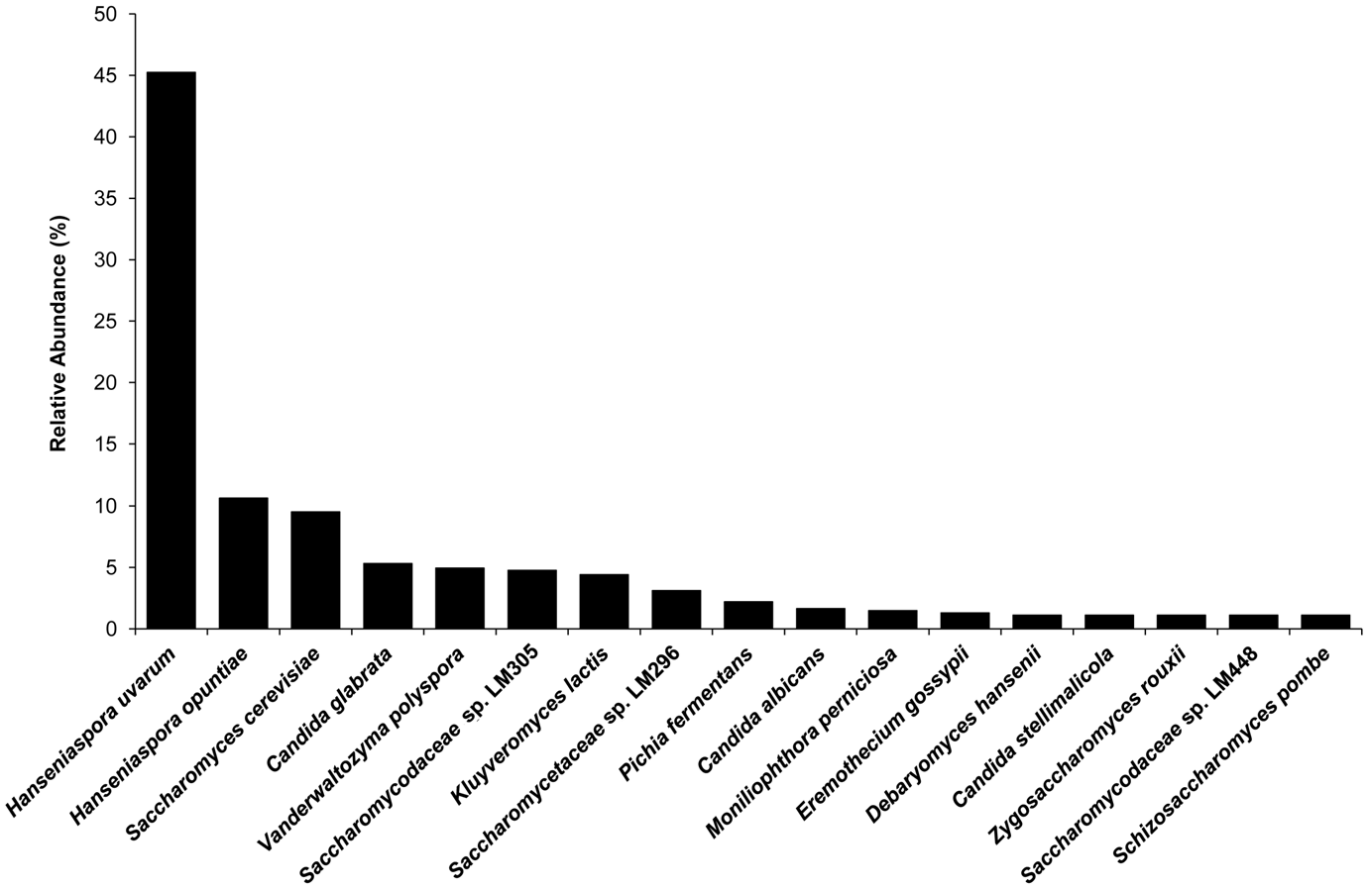
Diversity and richness of fungi on rank ‘species’.

Fungal rarefaction analysis was carried out with reads containing (part of) the internal transcribed spacer (ITS) regions ITS1 and/or ITS2. These regions were extracted from the data sets using the FungalITSextractor tool [49]. The reads containing a (partial) predicted ITS1-5.8S-ITS2 region were subsequently used in a BLASTN similarity search using the NCBI-nt database followed by their processing with MEGAN with the min support set to 2, the min score set to 100, and the top percent set to 1. At this stage, only hits within a fungal ITS region were taken into account to reduce false positives.

To assess whether all microbial species, both bacteria and fungi, of the spontaneous cocoa bean box fermentation sample under study were covered by the 454 pyrosequencing reads, rarefaction curves were constructed using the Analytic Rarefaction tool (version 1.3; www.uga.edu/strata/software/Software.html). The estimated numbers of genera associated with different sampling sizes of the environmental reads were expressed as OTUs. To avoid overestimation through misclassification, only genera that had an abundance of more than 0.01% of the data set were taken into account for bacterial rarefaction analysis.

**Table 2:** Comparison of different community composition analysis methods on rank species.

Species	Metagenomic DNA (%)	PCR-DGGE [14]	GTG_5_-PCR [14]	16S rRNA gene clone library** [7]
***Bacilli***				
*L. brevis*	0.5–11.4			
*L. casei*	0.0–0.9			
*L. fermentum*	9.3–96.8	X*	X*	X
*L. plantarum*	0.3–10.2	X	X	
*L. reuteri*	0.0–2.6	X		
*L. rhamnosus*	0.0–0.2			
*L. vaginalis*				X
*Lc. lactis*	0.0–0.9			
*Leuc. mesenteroides*	0.1–1.6			
*Leuc. pseudoficulneus* ***		X*		
*Leuc. pseudomesenteroides* ***		X		
*O. oeni*	0.0–1.8			
*P. acidilactici*			X	
*St. salivarius*			X*	
***α-Proteobacteria***				
*A. fabarum* ***			X	
*A. pasteurianus*	0.1–0.8	X	X*	X
*A. senegalensis* ***			X*	
*G. oxydans*	0.0–3.9		X	
*Ga. saccharivorans* ***			X	
***γ-Proteobacteria***				
*E. coli*	0.0–8.1			
*En. cloacae*	0.0–0.6			
*Er. amylovora*	0.0–0.8			
*Er. tasmaniensis*	0.0–5.6			
*K. pneumoniae*	0.1–1.9			
*Pe. carotovorum*	0.0–0.2			
*S. enterica*	0.0–2.2			
*T. citrea*		X*		
*T. ptyseos* ***		X*		

* Detected at 30 h.

** No analysis performed at 30 h.

*** Only phylogenetic marker gene(s) sequences available in databases.

Species are only considered as present in the ecosystem if they could be detected by all five taxonomic profiling tools. The relative species abundances, predicted by the different classification tools, are expressed as a range that represent the lowest and highest values obtained. For the other methods, the presence of a species is denoted by “X”. *A.*: *Acetobacter*; *E.*: *Escherichia*; *En.*: *Enterobacter*; *Er.*: *Erwinia*; *G.*: *Gluconobacter*; *Ga.*: *Gluconacetobacter*; *K.*: *Klebsiella*; *L.*: *Lactobacillus*; *Leuc.*: *Leuconostoc*; *Lc.*: *Lactococcus*; *O.*: *Oenococcus*; *P.*: *Pediococcus*; *Pe.*: *Pectobacterium*; *S.*: *Salmonella*; *St.*: *Streptococcus*; *T.*: *Tatumella*.

### Comparison of different taxonomic profiling tools to estimate the bacterial community composition

To assess the bacterial community composition of the sample under study, several software tools for taxonomic profiling were applied, using the combined data sets A and B. This was performed using both similarity- and composition-based software packages, designed for prokaryotic taxonomic profiling. All analyses were carried out on the taxonomic ranks phylum, class, order, family, and genus. To avoid software-dependent results, an OTU was only taken into account if it was predicted by at least five taxonomic profiling tools.

Applying a similarity-based analysis, tools based on extracting and classifying phylogenetic marker gene(s) (SmashCommunity, MetaPhyler) as well as tools based on classifying all environmental reads (SmashCommunity, MEGAN, CARMA) were used. For a phylogenetic marker gene-based analysis, SmashCommunity was used as described above. Analysis with MetaPhyler was performed using default parameters [50]. For an analysis based on all environmental reads, SmashCommunity and MEGAN were used as described above. CARMA (version 3) [51] was applied, based on a HMMER search using the Pfam database (version 24.0) [52].

Applying a composition-based analysis, RAIphy 1.0 [53] and PhymmBL [39] were used. For RAIphy, the binning threshold was set to 60 and a reference database was compiled based on the NCBI reference genomes. For PhymmBL, default parameters were used.

### Taxonomic profiling to estimate the fungal community composition

The fungal community composition was assessed using two approaches. In a first approach, MEGAN analysis of the results of a BLASTX search against the NCBI-nr database of data set A was performed with the min support set to 15 and the min score set to 100. The second approach was based on reads extracted from the combined data sets A and B and originating from the ITS region, as described above.

### Validation of the metagenomic approach

The results of the phylogenetic analysis of the metagenomic sequence data of the cocoa bean fermentation sample under study were compared with the results of former culture-dependent [(GTG)_5_-PCR genomic fingerprinting of isolates] and culture-independent community composition analysis methods (PCR-DGGE and/or 16S rRNA gene clone library sequencing of sample DNA) [7], [14]. A species was considered to be present in the ecosystem if it could be detected by all five prediction methods that were able to classify reads on rank species, namely SmashCommunity reference genomes, MEGAN, CARMA, RAIphy, and PhymmBL.

### Data availability

Sequence data from both GS FLX Titanium pyrosequencing runs were deposited in the NCBI Short Read Archive (SRA) under the accession number SRA049973.

## Results and Discussion

### Pyrosequencing and sequence data quality control

As the total bacterial diversity of a cocoa bean fermentation is limited [5], [6] and as only a few microbial species dominate the fermentation process [7], [17], [22], only half a PicoTiterPlate was initially used for pyrosequencing of the metagenomic DNA library of a Brazilian cocoa bean box fermentation sample. This pyrosequencing run resulted in 456,225 reads with an average length of 439 bases, which accounted for approximately 201-Mb sequence information (data set A; Table 1). To achieve a deeper coverage of the metagenomic DNA and to elucidate its whole complexity, the same DNA library was used for a second pyrosequencing run using a whole PicoTiterPlate, which yielded 1,248,151 reads with an average length of 441 bases and resulted in 552-Mb sequence information (data set B; Table 1). Data set B represented a 2.7-fold increase in coverage of the DNA sample compared to data set A. As the same DNA library was used for both pyrosequencing runs, the G+C contents of the environmental reads were approximately the same (≈49.6%). No decrease in G+C contents for longer read sizes were found for both pyrosequencing runs (Fig. S1), indicating no bias towards microorganisms with a lower G+C content [54]. The Bioconductor software package ShortRead indicated only a few exact duplicates (Table S1), which was confirmed by cd-hit-454 (data not shown). To avoid underestimation of certain microbial groups by removing natural duplicates instead of artificial duplicates, no reads were removed from the data sets.

### Bacterial and fungal community richness estimation through rarefaction analysis

The bacterial and fungal community richness of the reads of data set A was estimated using rarefaction curves based on taxonomic classification (Fig. S2). The rarefaction curves for bacteria indicated a gap between 16S rRNA gene-based methods and methods using all reads. Further, no saturation of the curves was reached with the 16S rRNA gene-based methods used. A rarefaction analysis of the combined data sets A and B revealed that saturation was reached, namely 36 OTUs for SmashCommunity 16S rRNA genes, 37 OTUs for SmashCommunity reference genomes, and 51 OTUs for MEGAN. This indicates that all bacterial members of the cocoa bean fermentation process sample were captured (Fig. 1).

The rarefaction curve for fungi based on data set A indicated that saturation was barely reached (Fig. S2). When using the combined data sets A and B, 755 reads containing the ITS1 and/or ITS2 rRNA gene regions were extracted. A rarefaction analysis of these sequence reads indicated saturation for the fungal communities of the cocoa bean fermentation ecosystem, namely 12 OTUs (Fig. 1).

### Comparison of different taxonomic profiling tools to estimate the bacterial community composition

The estimation of the bacterial community diversity varied (for all different taxonomic ranks) between the taxonomic profiling tools when they were evaluated independently (Table S2, rows A). However, when OTUs were only taken into account if they were predicted by five or more different taxonomic profiling tools, a more reliable overview of the members of the ecosystem was obtained (Table S2, rows B). For example, the results of both the similarity-based and composition-based methods were in accordance, when they were applied for high taxonomic ranks. Indeed, on rank phylum, all tools were consistent in predicting the amount of OTUs, *i.e.*, a high abundance of *Firmicutes* and *Proteobacteria* and a low abundance of *Actinobacteria* (data not shown). Analysis on rank class revealed that *Bacilli* were the most abundant, among which *Lactobacillales* was the predominant order, although there was a wide variety of orders between the tools used (from 49% in the case of PhymmBL to 83% in the case of SmashCommunity reference genomes; Fig. 2). Several orders within the class *γ-Proteobacteria* occurred, although this class was dominated by members of the order *Enterobacteriales*. Members of the classes *Actinobacteria* (orders *Actinomycetales* and *Bifidobacteriales*), *α-Proteobacteria* (dominated by order *Rhodospirillales*), and *Clostridia* were also present in the ecosystem under study, but to a lower extent. On rank order, the two composition-based classification tools (RAIphy and PhymmBL) predicted a wider diversity (especially within the class *γ-Proteobacteria*) than tools involving similarity-based methods. Indeed, both composition-based methods predicted the presence of different orders within the *γ-Proteobacteria*, whereas these orders were not, or only to a very low extent, found using similarity-based methods. This discrepancy could be explained by the fact that composition-based methods are able to classify reads without the availability of close relatives in sequence databases, whereas similarity-based methods do not classify these reads on lower ranks if sequence similarity is not above a set threshold [53]. However, the extended bacterial diversity within the *γ-Proteobacteria* predicted by composition-based methods (besides the diverse order *Enterobacteriales*) was consistent for the different methods used. For all taxonomic profiling tools, the family of *Lactobacillaceae* was the most abundant. Moreover, all tools identified *Lactobacillus* as the dominant genus, although large differences were found (from 46% for PhymmBL to 94% for MetaPhyler). *Lactobacillus* is indeed a widespread genus associated with cocoa bean fermentation processes [5], [6]. Analysis on rank genus of low-abundant members only, omitting the genus *Lactobacillus*, revealed differences in predicted OTUs between the classification tools (Fig. 3). Indeed, the three classification tools based on phylogenetic marker genes solely (both SmashCommunity 16S rRNA gene-based methods and MetaPhyler) predicted a restricted diversity compared with tools that took all reads into account. This difference could be ascribed to misclassifications of the latter tools, since binning of reads originating from non-phylogenetic marker genes is more prone to error; alternatively, it could be due to a failure of phylogenetic marker gene(s)-based methods [50]. The latter could originate from misclassifications due to absence of the phylogenetic marker gene sequences in the underlying database, or absence of phylogenetic marker genes in the data set because of insufficient sequencing. Hence, a phylogenetic analysis using only one taxonomic profiling tool should be interpreted carefully. However, predictions on rank genus by tools that took all reads into account were consistent, although some clear differences in abundance of these genera was seen. For instance, the PhymmBL tool classified 8.4% of the reads as *Escherichia*, which was a higher abundance compared with any other classification tool that classified only 0.0 to 1.4% of the reads as *Escherichia*. Database bias towards model bacteria such as *Escherichia coli* might explain this [55], [56].

Taxonomic profiling with the MEGAN package revealed the presence of 4,296 reads (0.25% of the total amount of reads) that originated from bacteriophages and that, therefore, were classified as viruses. These viral communities were dominated by *Lactobacillus* phages, although a few other bacterial hosts such as *Enterobacter* and *Klebsiella* were found as well (Fig. 4). As the DNA isolation method targeted bacteria and yeasts, it could be assumed that these reads were indeed from viral origin (such as prophages or remnants of bacteriophages), which were incorporated into the bacterial genomes, as lactobacilli often harbor phage DNA [57]–[59]. Indeed, a striking similarity between the dominant bacterial genera (*Lactobacillus*) and the dominant predicted bacteriophage hosts (*Lactobacillus*) was found, supporting the assumption that an interaction exists between bacterial hosts and the viral communities [60]. A restricted diversity within these phage DNA-associated sequences was found, as only members of the families *Siphoviridae* and *Myoviridae*, which belong to the order *Caudovirales*, were retrieved. Similarly, the viral communities of fermented shrimp, kimchi, and sauerkraut are dominated by bacteriophages belonging to the viral order *Caudovirales* [61]. This is the first report on the occurrence of bacteriophages of lactobacilli in a cocoa bean fermentation sample. However, it is well known that bacteriophages are associated with LAB involved in food fermentation processes [62], [63].

### Taxonomic profiling of the fungal community composition

Fungal community composition analysis of data set A classified only 2,032 reads (0.16%) within the kingdom *Fungi*. Almost all reads were classified on high taxonomic ranks. Only 268 out of the 2,032 reads could be classified on genus or species level, the latter being classified as *Hanseniaspora uvarum, Kluyveromyces lactis*, *Lachancea thermotolerans*, *Pichia angusta*, *Saccharomyces cerevisiae*, and *Zygosaccharomyces rouxii*. This indicates that a BLASTX-based MEGAN analysis of a whole-community metagenomic data set was not suitable to classify the reads on a low taxonomic rank. However, a combination of extracting reads containing a (partial) ITS region and a subsequent BLASTN similarity search was able to classify a total of 755 reads on rank species (Fig. 5). The present metagenomic analysis indicates that the most prevailing yeast was *H. uvarum*, followed by *Hanseniaspora opuntiae*, and *S. cerevisiae*, which accounted for 45.2%, 10.6%, and 9.5% of all yeast DNA, respectively. These species are commonly associated with cocoa bean fermentation processes [20], [64]. Further, other species commonly occurring during cocoa bean fermentations were found in the current sample as well, such as *Candida glabrata*, *K. lactis*, *Pichia fermentans*, *Debaryomyces hansenii*, *Candida stellimalicola*, *Schizosaccharomyces pombe*, and species of the families *Saccharomycodaceae* and *Saccharomycetaceae*. In addition, fungal species that were not yet associated with cocoa bean fermentations were identified. For instance, *Vanderwaltozyma polyspora* is a yeast species previously isolated from a soil ecosystem [65] and *Z. rouxii* has been reported in miso and soy sauce fermentations [66]. *Moniliophthora perniciosa* and *Eremothecium gossypii*, both plant pathogenic filamentous fungi, and the human pathogenic *Candida albicans* were found as well, but their identification could be the result of database bias towards pathogenic fungal species [55], [56]. However, as *M. perniciosa* causes witches' broom disease of cocoa trees [67], it is not surprising that this species was present in fermenting cocoa pulp-bean mass.

### Validation of the metagenomic approach

A comparison of the results of the phylogenetic analysis using different computational methods with former results of culture-dependent and culture-independent community composition analyses revealed that the metagenomic approach was able to retrieve most of the previously identified members (Table 2). This was even the case on rank species, which is generally regarded as inaccurate [68]. *Lactobacillus fermentum* and *A. pasteurianus* were identified as the prevailing LAB and AAB species, respectively, which was in accordance with 16S rRNA gene-PCR-DGGE and (GTG)_5_-PCR fingerprinting analyses of this 30-h fermentation sample [7], [14]. This underlines the functional role of both species during cocoa bean fermentation [6], [69]. Also, *L. plantarum, Lactobacillus reuteri*, and *G. oxydans* were identified by metagenomic sequencing of the 30-h fermentation sample, whereas these species were not found by 16S rRNA gene-PCR-DGGE and/or (GTG)_5_-PCR analysis. Further, the metagenomic analysis revealed the presence of several bacterial species, which were not detected in this fermentation sample by culture-dependent and/or culture-independent analysis. This included opportunistic members of the cocoa bean fermentation process, such as *E. tasmaniensis*, *Lactobacillus brevis*, *Lactobacillus casei*, *Lactococcus lactis*, *Leuconostoc mesenteroides*, and *Oenococcus oeni* [3], [7], [12], [18], [70]–[72]. Additionally, some bacterial species were found that were not yet detected during cocoa bean fermentation processes, such as *Lactobacillus rhamnosus*, an intestinal inhabitant [73]. Further, *Pectobacterium carotovorum* and *Erwinia amylovora* are phytopathogens, causing potato rot diseases and wilt diseases on *Rosaceae*, respectively [74]. The occurrence of *Escherichia coli*, *Salmonella enterica*, *Klebsiella pneumoniae*, and *Enterobacter cloacae*, might be unexpected, although contamination with gastro-intestinal (pathogenic) bacteria may occur. However, as only relatively few reads were classified within these species (<1%), their presence could be the result of an overestimation due to a bias towards (pathogenic) model bacteria in the databases used [55], [56]. In contrast, other species such as *Fructobacillus pseudoficulneus*, *Acetobacter senegalensis*, and *Tatumella ptyseos* were not retrieved using the metagenomic approach. This was probably due to the lack of sequence information of these species, for which only one or a few phylogenetic marker gene(s) are available, in public databases that were used to perform the taxonomic profiling. Further, it could be due to differences in homogeneity in sample material in the case these species display a low abundance at the particular time point investigated.

Former results indicated that the prevailing yeast in this cocoa bean fermentation sample is a *Hanseniaspora* species, most likely *H. opuntiae* [64]. However, this species could not be distinguished from *H. uvarum* and *Hanseniaspora guilliermondii* through 26S rRNA gene-PCR-DGGE [64]. In contrast, the metagenomic sequencing approach of the present study revealed that *H. uvarum* is the prevailing yeast at this time point. Indeed, using reads originating from the ITS region, it was possible to differentiate between *H. uvarum* and *H. opuntiae*. Hence, it is likely to assume that *H. uvarum*, *H. opuntiae*, and *S. cerevisiae* are the prevailing yeasts during cocoa bean fermentation. Additionally, whereas only a few yeast species were detected before, a wide fungal diversity was found in this sample using a metagenomic approach.

### Conclusions

This study is the first report on the taxonomic analysis of a cocoa bean fermentation sample using a metagenomic approach, *i.e.*, 454 pyrosequencing of whole-community DNA. It was shown that this approach, when applying two pyrosequencing runs to obtain a high depth of coverage, was suitable to reveal both dominant and rare bacterial and fungal members of the cocoa bean fermentation ecosystem at a certain time point and to identify associated bacteriophages. However, this approach does not provide information about the community dynamics throughout the whole fermentation process. A combination of different similarity-based and composition-based methods, including both phylogenetic marker gene(s)-based analysis as well as methods using all available sequence information, appears to be necessary to obtain a credible view on the microbial community diversity of a complex microbial ecosystem, such as the cocoa bean fermentation process. Dominant species were *H. uvarum, H. opuntiae, S. cerevisiae, L. fermentum*, and *A. pasteurianus*, which was in accordance with former culture-dependent and culture-independent community analysis methods and underlines their importance in cocoa bean fermentations. In addition, sequence reads associated with viral communities were found, representing only members of the families *Myoviridae* and *Siphoviridae*, with *Lactobacillus* as the dominant microbial host, which is in accordance with the microbial phylogenetic analysis. These results indicate the superiority of metagenomic sequencing over previously used techniques for a phylogenetic characterization of complex matrices such as that involved in the cocoa bean fermentation process. The wider diversity retrieved in the present study is of importance to generate further insights into the functional roles of bacteria, fungi, and bacteriophages during cocoa bean fermentation, which is of great importance to select an appropriate starter culture for homogeneous, fast, and successfully controlled processes [75]–[77].

## Supporting Information

Figure S1 **Distribution of the average G+C content as a function of read length.** Two pyrosequencing data sets were used, which were the result of a pyrosequencing run using half a PicoTiterPlate and a complete PicoTiterPlate.
(TIF)

Figure S2 **Rarefaction analysis of the genera found with data set A.** The rarefaction curves represent an estimation of the number of genera associated with different sampling sizes. As the results of the two 16S rRNA gene-based methods of the SmashCommunity platform were similar, only one method (based on the similarity with a 16S rRNA gene sequence database of the SmashCommunity platform) is shown. As the plateau phase of the SmashCommunity reference genomes platform and MEGAN was reached at 25,000 reads, the X-axis is limited to this number of reads.
(TIF)

Table S1 **Duplicate reads.** Data set A refers to the sequencing run using two regions of a four-region gasket (half a PicoTiterPlate); data set B refers to the sequencing run of a complete PicoTiterPlate.
(DOC)

Table S2 **Bacterial community diversity estimations for the eight taxonomic profiling tools used.** For each of the taxonomic profiling tools used, the numbers in row A refer to the originally estimated OTUs per rank; the numbers in row B refer to a subsection of the OTUs in row A that were also estimated by at least four other taxonomic profiling tools. The numbers between brackets depict the percentage of reads used to estimate the number of OTUs in row A that are included by the OTUs in row B. ‘SmashCommunity RG’ depicts SmashCommunity reference genomes, ‘SmashCommunity 16S (1)’ depicts the SmashCommunity 16S rRNA gene-based method using the meta_rrna approach, ‘SmashCommunity 16S (2)’ depicts the SmashCommunity 16S rRNA gene-based method using the 16S rRNA gene sequence database approach.
(DOC)
