# Supplementary figures and images for: Phylogenetic Analysis of a Spontaneous Cocoa Bean Fermentation Metagenome Reveals New Insights into Its Bacterial and Fungal Community Diversity
Source: PLoS One. 2012 May 29;7(5):e38040. doi: 10.1371/journal.pone.0038040 (PMC3362557; doi:10.1371/journal.pone.0038040)

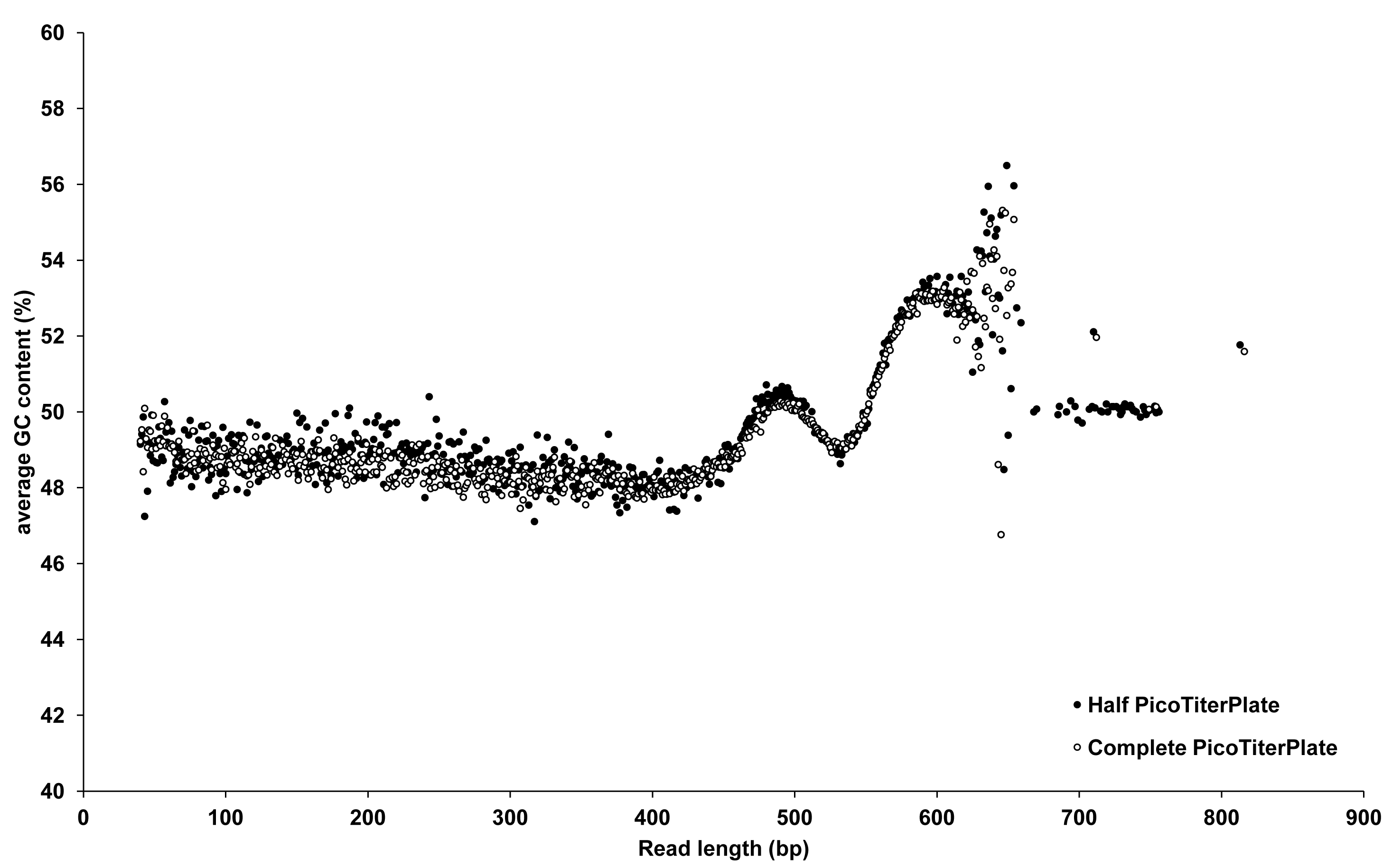

Supplement: Figure S1 — Distribution of the average G+C content as a function of read length. Two pyrosequencing data sets were used, which were the result of a pyrosequencing run using half a PicoTiterPlate and a complete PicoTiterPlate. (TIF) [file pone.0038040.s001.tif]

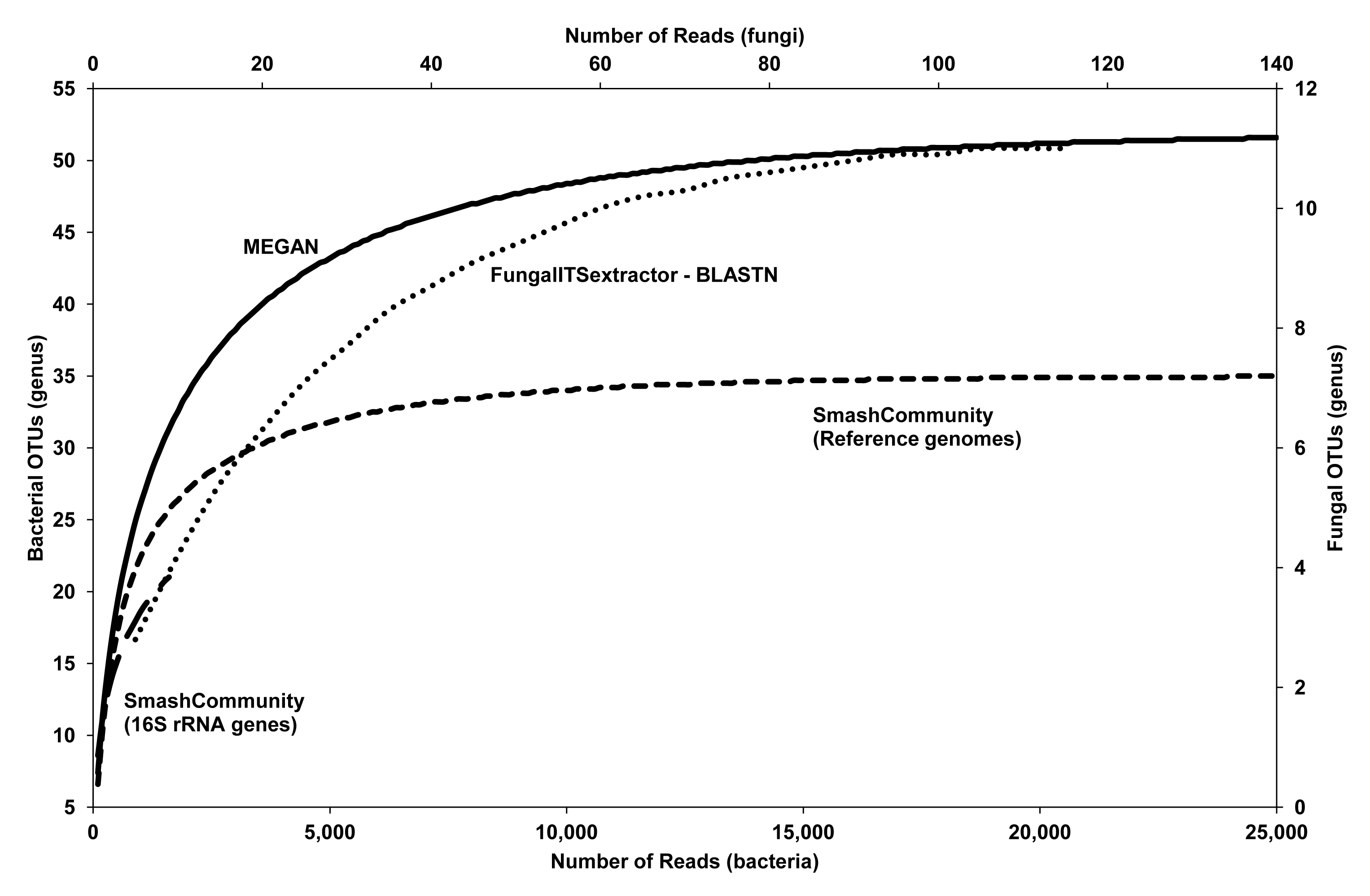

Supplement: Figure S2 — Rarefaction analysis of the genera found with data set A. The rarefaction curves represent an estimation of the number of genera associated with different sampling sizes. As the results of the two 16S rRNA gene-based methods of the SmashCommunity platform were similar, only one method (based on the similarity with a 16S rRNA gene sequence database of the SmashCommunity platform) is shown. As the plateau phase of the SmashCommunity reference genomes platform and MEGAN was reached at 25,000 reads, the X-axis is limited to this number of reads. (TIF) [file pone.0038040.s002.tif]
