# Supplementary material for: Phylogenetic Analysis of a Spontaneous Cocoa Bean Fermentation Metagenome Reveals New Insights into Its Bacterial and Fungal Community Diversity
Source: PLoS One. 2012 May 29;7(5):e38040. doi: 10.1371/journal.pone.0038040 (PMC3362557; doi:10.1371/journal.pone.0038040)
Supplement: Table S1 — Duplicate reads. Data set A refers to the sequencing run using two regions of a four-region gasket (half a PicoTiterPlate); data set B refers to the sequencing run of a complete PicoTiterPlate. (DOC) [file pone.0038040.s003.doc]

| **Data set A region 1** | | **Data set A region 2** | | **Data set B region 1** | | **Data set B region 2** | |
| --- | --- | --- | --- | --- | --- | --- | --- |
| **# occurrence** | **# reads** | **# occurrence** | **# reads** | **# occurrence** | **# reads** | **# occurrence** | **# reads** |
| 1 | 238022 | 1 | 215011 | 1 | 602574 | 1 | 638875 |
| 2 | 860 | 2 | 462 | 2 | 1528 | 2 | 1484 |
| 3 | 46 | 3 | 37 | 3 | 54 | 3 | 49 |
| 4 | 3 | 4 | 2 | 4 | 3 | 4 | 4 |
| 5 | 1 | 5 | 3 | 5 | 5 | 11 | 2 |
| 6 | 1 | 6 | 2 | 6 | 3 |  |  |
| 8 | 2 | 7 | 1 | 9 | 1 |  |  |
| 11 | 1 | 12 | 1 | 11 | 1 |  |  |
| 18 | 1 | 16 | 1 |  |  |  |  |
| 29 | 1 | 24 | 1 |  |  |  |  |
| 39 | 1 | 38 | 1 |  |  |  |  |
