# Supplementary material for: Phylogenetic Analysis of a Spontaneous Cocoa Bean Fermentation Metagenome Reveals New Insights into Its Bacterial and Fungal Community Diversity
Source: PLoS One. 2012 May 29;7(5):e38040. doi: 10.1371/journal.pone.0038040 (PMC3362557; doi:10.1371/journal.pone.0038040)
Supplement: Table S2 — Bacterial community diversity estimations for the eight taxonomic profiling tools used. For each of the taxonomic profiling tools used, the numbers in row A refer to the originally estimated OTUs per rank; the numbers in row B refer to a subsection of the OTUs in row A that were also estimated by at least four other taxonomic profiling tools. The numbers between brackets depict the percentage of reads used to estimate the number of OTUs in row A that are included by the OTUs in row B. ‘SmashCommunity RG’ depicts SmashCommunity reference genomes, ‘SmashCommunity 16S (1)’ depicts the SmashCommunity 16S rRNA gene-based method using the meta_rrna approach, ‘SmashCommunity 16S (2)’ depicts the SmashCommunity 16S rRNA gene-based method using the 16S rRNA gene sequence database approach. (DOC) [file pone.0038040.s004.doc]

|  |  | **Rank** | | | | | |
| --- | --- | --- | --- | --- | --- | --- | --- |
|  |  | **Phylum** | **Class** | **Order** | **Family** | **Genus** | **Species** |
| **MetaPhyler** | A | 5 | 5 | 16 | 4 | 9 | - |
|  | B | 4 (99.94) | 5 (100.00) | 4 (99.62) | 4 (100.00) | 9 (100.00) | - |
| **Smash** | A | 5 | 13 | 21 | 23 | 40 | 37 |
| **Community RG** | B | 4 (99.99) | 6 (99.42) | 16 (99.36) | 16 (99.39) | 30 (99.42) | 18 (98.74) |
| **Smash** | A | 3 | 4 | 6 | 21 | 47 | - |
| **Community 16S (1)** | B | 3 (100.00) | 4 (100.00) | 6 (100.00) | 7 (99.93) | 19 (90.97) | - |
| **Smash** | A | 3 | 4 | 6 | 22 | 26 | - |
| **Community 16S (2)** | B | 3 (100.00) | 4 (100.00) | 6 (100.00) | 8 (99.94) | 20 (92.73) | - |
| **MEGAN** | A | 5 | 9 | 21 | 34 | 51 | 104 |
|  | B | 3 (99.94) | 7 (99.96) | 18 (99.97) | 22 (99.83) | 30 (99.17) | 18 (92.82) |
| **CARMA** | A | 11 | 12 | 30 | 44 | 86 | 278 |
|  | B | 4 (99.76) | 7 (99.80) | 19 (99.75) | 22 (98.69) | 29 (96.16) | 18 (52.07) |
| **RAIphy** | A | 24 | 38 | 79 | 139 | 266 | 409 |
|  | B | 4 (91.85) | 7 (90.17) | 19 (87.54) | 22 (81.06) | 28 (73.51) | 18 (62.39) |
| **PhymmBL** | A | 25 | 44 | 68 | 142 | 286 | 475 |
|  | B | 4 (94.67) | 7 (94.07) | 19 (90.85) | 22 (85.45) | 28 (76.34) | 18 (61.28) |
